# Predication of oxygen requirement in COVID-19 patients using dynamic change of inflammatory markers: CRP, hypertension, age, neutrophil and lymphocyte (CHANeL)

**DOI:** 10.1038/s41598-021-92418-2

**Published:** 2021-06-22

**Authors:** Eunyoung Emily Lee, Woochang Hwang, Kyoung-Ho Song, Jongtak Jung, Chang Kyung Kang, Jeong-Han Kim, Hong Sang Oh, Yu Min Kang, Eun Bong Lee, Bum Sik Chin, Woojeung Song, Nam Joong Kim, Jin Kyun Park

**Affiliations:** 1 Division of Rheumatology, Department of Internal Medicine, Uijeongbu Eulji Medical Center, Eulji University School of Medicine, Uijeongbu-Si, Gyeonggi-do South Korea; 2 Hanyang Biomedical Research Institute, Hanyang University, Seoul, South Korea; 3 Division of Infectious Diseases, Department of Internal Medicine, Seoul National University Bundang Hospital, Seongnam, South Korea; 4 Division of Infectious Diseases, Department of Internal Medicine, Seoul National University College of Medicine, 101, Daehak-ro, Jongno-gu, Seoul, 03080 South Korea; 5 Division of Infectious Diseases, Department of Internal Medicine, Armed Forces Capital Hospital, Seongnam-Si, Gyeonggi-do South Korea; 6 Department of Infectious Diseases, Myongji Hospital, Goyang, Gyeonggi-do South Korea; 7 Department of Medical Education, Seoul National University College of Medicine, Seoul, South Korea; 8 Division of Rheumatology, Department of Internal Medicine, Seoul National University Hospital and Seoul National University College of Medicine, 101, Daehak-ro, Jongno-gu, Seoul, 03080 South Korea; 9 Division of Infectious Diseases, Department of Internal Medicine, National Medical Center, Seoul, South Korea; 10 Department of Medicine, Major in Medical Genetics, Graduate School, Hanyang University, Seoul, South Korea

**Keywords:** Infectious diseases, Inflammation

## Abstract

The objective of the study was to develop and validate a prediction model that identifies COVID-19 patients at risk of requiring oxygen support based on five parameters: C-reactive protein (CRP), hypertension, age, and neutrophil and lymphocyte counts (CHANeL). This retrospective cohort study included 221 consecutive COVID-19 patients and the patients were randomly assigned randomly to a training set and a test set in a ratio of 1:1. Logistic regression, logistic LASSO regression, Random Forest, Support Vector Machine, and XGBoost analyses were performed based on age, hypertension status, serial CRP, and neutrophil and lymphocyte counts during the first 3 days of hospitalization. The ability of the model to predict oxygen requirement during hospitalization was tested. During hospitalization, 45 (41.8%) patients in the training set (n = 110) and 41 (36.9%) in the test set (n = 111) required supplementary oxygen support. The logistic LASSO regression model exhibited the highest AUC for the test set, with a sensitivity of 0.927 and a specificity of 0.814. An online risk calculator for oxygen requirement using CHANeL predictors was developed. “CHANeL” prediction models based on serial CRP, neutrophil, and lymphocyte counts during the first 3 days of hospitalization, along with age and hypertension status, provide a reliable estimate of the risk of supplement oxygen requirement among patients hospitalized with COVID-19.

## Introduction

Since the first case in Wuhan, China, in December 2019, the novel coronavirus disease 2019 (COVID-19) has spread rapidly world-wide. The clinical course and outcome of COVID-19 varies markedly from asymptomatic and mild, to critical and lethal. While young people without underlying comorbidities tend to have asymptomatic or mild disease, elderly patients and those with comorbidities (such as cardiovascular disease, diabetes mellitus, hypertension, chronic lung disease, cancer, and chronic kidney disease) are at an increased risk of death from respiratory failure and sepsis^1–4^.

In the absence of effective and/or preventive treatments, the outcome for critically ill COVID-19 patients depends on the availability of supportive intensive medical care^5^. The rapid spread of COVID-19 as a global pandemic has brought extraordinary challenges to the healthcare system. When the healthcare system is overwhelmed by a massive influx of patients, mortality increases^6^. In the face of limited resources, it is critical to reliably identify COVID-19 patients who require close monitoring and intensive care, including supplementary oxygen and/or mechanical ventilation, while those patients with a good prognosis can be monitored at home or managed at a living and treatment center^7^. A prediction model that can identify patients at high risk of respiratory failure at an early stage will help optimal allocation of limited resources.

During the early stage of COVID-19 infection, immunologic responses differ between survivors and non-survivors^2^. Since clinical and laboratory parameters (especially inflammatory markers) are subject to dynamic change, trends (i.e., time-series measurements) might better capture onset of a potentially lethal hyper-inflammatory immune response, which is associated with a severe clinical course and a poor outcome^8^.

Here, we aimed to construct a prediction model that identifies COVID-19 patients at high risk of developing respiratory failure. Based on our previous findings, we a priori selected five parameters: CRP, hypertension status, age, and neutrophil and lymphocyte counts (CHANeL). We hypothesized that the pattern of CRP, and neutrophil and lymphocyte counts during the first 3 days of hospitalization are predictive of the type (e.g., hyper-inflammatory) of inflammatory response likely to occur during the course of infection. We constructed several prediction models including a logistic regression, logistic LASSO regression, a Random Forrest model, a Support Vector Machine, and XGBoost. We found that the logistic LASSO regression model showed high sensitivity and specificity for identifying patients with COVID-19 who are at high risk of respiratory failure during hospitalization.

## Results

### Baseline characteristics

**Figure 1:**
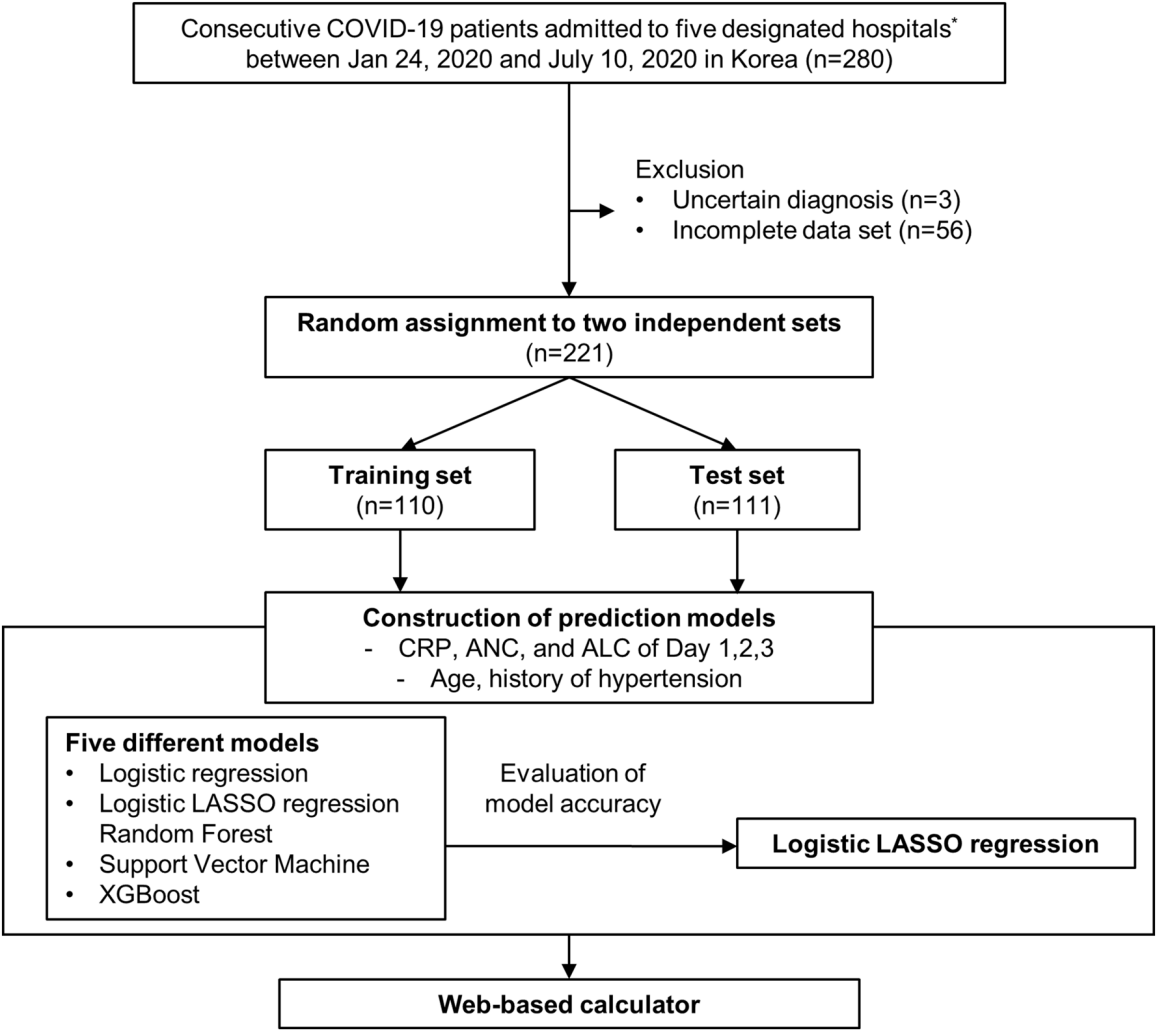
Study design and patient flow. ^*^National Medical Center (n = 128), Seoul National University Hospital (n = 46), Armed Forces Capital Hospital (n = 41), Myongji Hospital (n = 40), and Seoul National University Bundang Hospital (n = 25). ALC, absolute lymphocyte count; ANC, absolute neutrophil count; CRP, C-reactive protein.

Between January 24, 2020 and July 10, 2020, 280 consecutive patients with COVID-19 were enrolled. After excluding patients with an unclear diagnosis (n = 3) and missing data (n = 56), 221 patients were assigned randomly to a training set (n = 110) or a test set (n = 111) (Fig. 1). The mean age of the patients in the training and test sets was 56.0 and 55.0 years, respectively, and 58.2% and 65.8%, respectively, were male. The clinical characteristics of the patients in the training and test sets at the time of admission are shown in Table 1. There was no difference in baseline pulse oximetric saturation/fraction of inspired oxygen (SpO_2_/FiO_2_) ratio and other clinical and laboratory features between the groups. The prevalence of hypertension, diabetes and chronic kidney disease were similar in the training set and the test set (Table 1.) Forty-six patients (41.8%) in the training set and 41 (36.9%) in the test set required supplementary oxygen during hospitalization. The patients received supplementary oxygen therapy when clinically indicated (SpO_2_ < 92% or any shortness of breath on room air). The mode of oxygen administration was subject to change based on the patient’s condition as described in Supplementary Table S2.

**Table 1:** Clinical characteristics of the patients in the cohort.

	Training set (n = 110)	Test set (n = 111)	*P*-value
Male, n (%)	64 (58.2)	73 (65.8)	0.306
Age (years)	56.0 [37.0; 68.0]	55.0 [34.5; 67.5]	0.751
**Comorbidities, n (%)**			
Hypertension	25 (22.7)	21 (18.9)	0.595
Diabetes	21 (19.1)	12 (10.8)	0.124
Chronic kidney disease	5 (4.5)	3 (2.7)	0.497
Time from symptom onset to admission (days)	5.0 [1.0; 10.0]	5.0 [2.0; 10.0]	0.292
SpO_2_/FiO_2_ ratio	457.1 [339.3; 466.7]	461.9 [447.6; 461.9]	0.408
**Laboratory parameters**			
White blood cell count (4.0–10.0) (10^3^/µL)	5.6 [4.1; 7.6]	5.3 [ 4.1; 6.7]	0.367
Neutrophils (50–75) (%)	69.2 [59.0; 77.6]	65.1 [52.9; 73.4]	0.040
Lymphocytes (20–40) (%)	20.5 [13.6; 30.2]	24.5 [16.6; 34.5]	0.029
Absolute neutrophil count (1,500–8,000) (/µL)	3612.0 [2511.6; 5859.7]	3535.2 [2252.8; 4935.7]	0.112
Absolute lymphocyte count (1,000–4,800) (/µL)	1080.3 [773.8; 1498.1]	1140.3 [875.0; 1660.1]	0.097
Hemoglobin (g/dL)	13.3 [12.1; 15.2]	14.1 [12.6; 15.4]	0.080
Platelet (10^3^/µL)	207.0 [166.0; 269.0]	212.5 [169.0; 255.0]	0.922
C-reactive protein (0–0.5) (mg/dL)	1.8 [0.2; 6.3]	1.0 [0.2; 5.8]	0.220
Aspartate transaminase (IU/mL)	31.0 [24.0; 47.0]	28.0 [22.0; 40.0]	0.165
Alanine transaminase (IU/mL)	23.5 [15.0; 39.5]	26.0 [17.0; 37.0]	0.754
Blood urea nitrogen (mg/dL)	12.9 [9.0; 16.0]	13.0 [9.0; 16.5]	0.795
Creatinine (mg/dL)	0.8 [0.6; 0.9]	0.8 [0.7; 0.9]	0.823
Prothrombin time (INR)	1.0 [1.0; 1.1]	1.0 [1.0; 1.1]	0.624
Activated partial thromboplastin time (s)	35.6 [33.3; 40.0]	35.8 [33.4; 39.9]	1.000
Albumin (g/dL)	4.0 [3.6; 4.5]	4.2 [3.8; 4.7]	0.097
**Supplement oxygen during hospitalization, n (%)**	46 (41.8)	41 (36.9)	0.545
High flow nasal cannula	17 (15.5)	11 (9.9)	0.300
Invasive mechanical ventilation	13 (11.8)	6 (5.4)	0.144
Extracorporeal membrane oxygenation	3 (2.7)	2 (1.8)	0.683
Death, n (%)	2 (1.8)	4 (3.6)	0.683

Normally distributed values are expressed as the mean (standard deviation), and non-normally distributed values are expressed as the median [interquartile range].

### Prediction models

**Figure 2:**
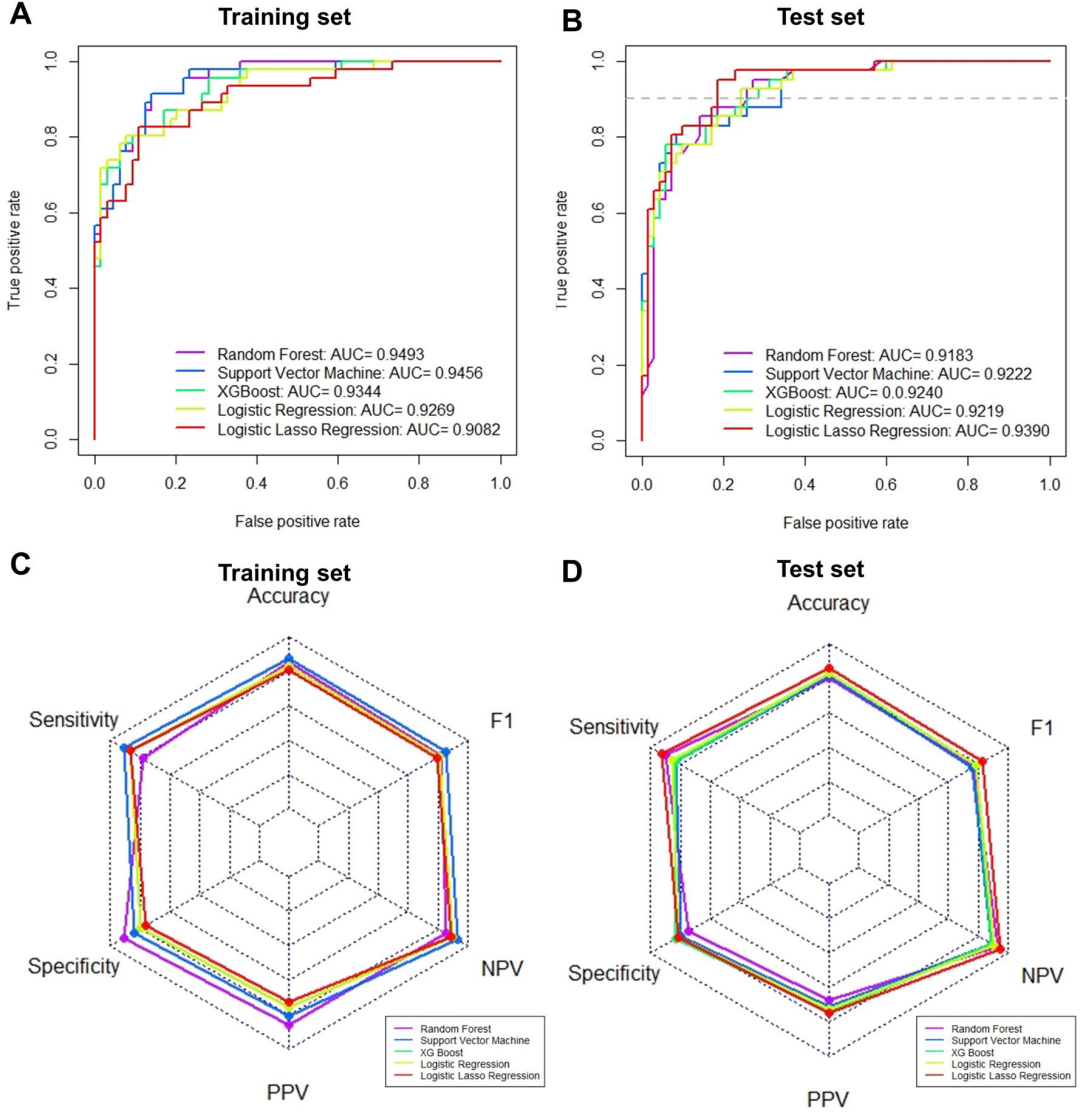
Receiver operating characteristic curve (ROC) and radar plot showing the performance of five different prediction models. (**A**) ROC curve for the training set. (**B**) ROC curve for the test set. (**C**) Radar plot for the training set. (**D**) Radar plot for the test set. AUC, area under the curve; NPV, negative predictive value; PPV, positive predictive value.

We developed multivariate risk prediction models to assess the primary outcome (i.e., requirement of supplementary oxygen during hospitalization) based on five variables. All five models showed a high AUC > 0.9 for the training set and test set. Among them, the logistic LASSO regression model showed the highest AUC for the test set (Fig. 2A,B).

### Sensitivity and specificity of the prediction models

The probability cut-off for each model was set at 0.3 to increase the sensitivity (at the cost of specificity). Sensitivity, specificity, predictive values, and accuracy of the predictor models for both the training and test set were high (Table 2 and Fig. 2C,D). Among the test models, the logistic LASSO regression model showed the highest sensitivity (0.927) and specificity (0.814) for the test set. All models had a high negative predictive value (NPV). When the probability cut-off value was set to > 0.4, the specificity (for the training and test set) and accuracy (for the training set) improved, but the sensitivity decreased.

**Table 2:** Sensitivity, specificity, positive predictive value, negative predictive value, and accuracy of the models for the training and test set (probability cut-off =  > 0.3, > 0.4, or > 0.5).

	Training set					Test set				
	Se	Sp	PPV	NPV	Accuracy	Se	Sp	PPV	NPV	Accuracy
**Cutoff > 0.3**										
Logistic regression	0.870	0.797	0.755	0.895	0.827	0.854	0.814	0.729	0.905	0.829
Logistic LASSO regression	0.870	0.766	0.727	0.891	0.809	0.927	0.814	0.729	0.905	0.829
Support Vector Machine	0.913	0.844	0.808	0.931	0.873	0.829	0.800	0.708	0.889	0.811
XG Boost	0.870	0.797	0.755	0.895	0.827	0.829	0.829	0.739	0.829	0.829
Random Forest	0.783	0.906	0.857	0.853	0.855	0.902	0.743	0.673	0.929	0.802
**Cutoff > 0.4**										
Logistic regression	0.804	0.891	0.841	0.864	0.855	0.781	0.829	0.727	0.866	0.811
Logistic LASSO regression	0.804	0.891	0.841	0.864	0.855	0.829	0.871	0.791	0.897	0.856
Support Vector Machine	0.783	0.906	0.857	0.853	0.855	0.829	0.871	0.791	0.897	0.856
XG Boost	0.804	0.891	0.841	0.864	0.855	0.781	0.857	0.762	0.870	0.829
Random Forest	0.717	0.938	0.892	0.822	0.846	0.854	0.814	0.729	0.905	0.829
**Cutoff > 0.5**										
Logistic regression	0.783	0.938	0.900	0.857	0.873	0.781	0.886	0.800	0.873	0.847
Logistic LASSO regression	0.696	0.906	0.842	0.806	0.818	0.805	0.929	0.868	0.890	0.883
Support Vector Machine	0.674	0.938	0.886	0.800	0.827	0.781	0.914	0.842	0.877	0.865
XG Boost	0.717	0.938	0.892	0.822	0.846	0.781	0.900	0.821	0.875	0.856
Random Forest	0.630	0.953	0.906	0.782	0.818	0.805	0.857	0.767	0.882	0.838

NPV, negative predictive value; PPV, positive predictive value; Se, sensitivity; Sp, specificity.

### Estimated predictive value of the CHANeL parameters

The individual contribution of each of the five predictors was estimated (Supplementary Table S1). In the logistic LASSO regression model, the CRP value on Day 3 had the highest impact in all five models, whereas the CRP level on Days 1 and 2 played less of a role. In the Random Forest model, variables of the first 3 days were important.

### Construction of a calculator

**Figure 3:**
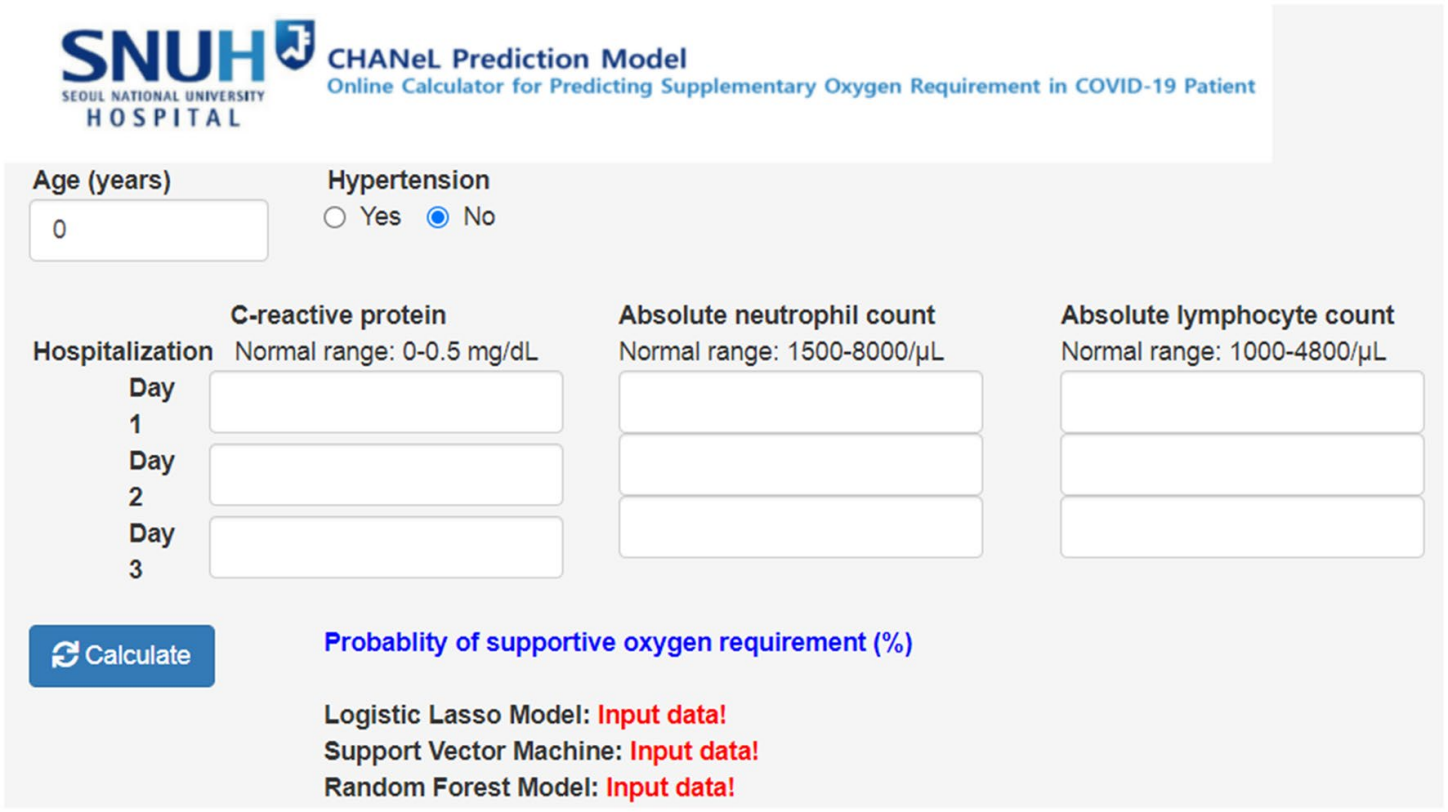
CHANeL prediction model.

An online calculator based on the logistic LASSO regression model and the Random Forest model using the CHANeL predictors was developed to calculate the risk score for a hospitalized patient with COVID-19 requiring supplementary oxygen during hospitalization (http://166.104.118.164:3838/chanel/) (Fig. 3).

## Discussion

To the best of our knowledge, this study was the first attempt to include pattern of the routine inflammatory markers during the early stage of disease in model to predict requirement for supplementary oxygen (i.e., respiratory failure) among hospitalized patients with COVID-19. All models based on CHANeL (age, hypertension, serial CRP, and neutrophil and lymphocyte counts during the first 3 days of hospitalization) showed high accuracy.

The unique strength of the CHANeL prediction models is the hypothesis-driven a priori selection of the five predictors. We showed previously that a hyper-inflammatory immune response, characterized by high CRP levels, high neutrophil counts, and low lymphocyte counts, was associated with a requirement for supplementary oxygen support and a worse outcome, whereas a normal inflammatory response, characterized by minimal elevation of CRP, a normal neutrophil count, and a normal lymphocyte count, was associated with an excellent outcome^8^. The inflammatory markers were similar on day of admission and started to differ between patients who required supplementary oxygen and those who did not in the first few days of illness. The difference become prominent in the second week of hospitalization. Thus, the dynamic changes (i.e., patterns) in common inflammatory markers (CRP, and neutrophil and lymphocyte counts) in early disease course were strongly associated with overall inflammatory response and clinical severity of COVID19^8^. A retrospective study of 136 COVID-19 patients showed that initial clinical and laboratory characteristics at admission were not predictive of this deterioration, further supporting that parameters measured at a single time point might not be sensitive enough to identify patients at risk^9^. To increase the model accuracy, we included two known demographic risk factors (age and hypertension), which have been identified consistently as demographic characteristics associated with a worse outcome^2,5^.

Numerous laboratory parameters have been suggested as risk factors for a worse outcome of COVID-19 disease; these include increased neutrophil counts, decreased lymphocyte counts (and, thus, the neutrophil/lymphocyte ratio), elevated CRP levels, and elevated d-dimer levels^2,4,10–13^. Others identified serum hydrogen sulfide and soluble urokinase plasminogen activator receptor as potential predictors for severe pneumonia in COVID-19^14,15^. Furthermore, blood levels of interleukin (IL)-1, IL-6, IL-8, and tumor necrosis factor (TNF) are associated with severity and prognosis of COVID-19^16^. TNF and IL-6 drive hepatic synthesis of CRP, whereas IL-8 increases neutrophil recruitment. Therefore, the levels of these cytokines are reflected indirectly by CRP levels and neutrophil counts in the CHANeL model. Liang et al. developed a clinical risk score to predict the probability of developing a critical illness. The score system was based on ten variables measured at admission, all of which were selected from an initial 72 candidates^17^. Similarly, the other prediction models for severe COVID-19 such as CANPT score or CMR tool are based on scoring of numerous parameters at admission^18,19^. By contrast, the CHANeL model is based on the hypothesis that the inflammatory response ultimately determines the clinical course of COVID-19. Since clinical manifestations such as hemoptysis, dyspnea, chest X-ray abnormalities, and mental status change, and laboratory parameters are considered to be the result (not the cause) of a systemic inflammatory response to viral infection, they were not included. Despite, or because of, its simplicity, the performance of the CHANeL-based prediction models was high; all models had an AUC of > 0.9 (Fig. 1). The five different models were indirectly compared with regards to sensitivity, specificity, positive predictive value, negative predictive value and accuracy, and the logistic LASSO model and the Random Forrest Model showed the best sensitivity and specificity (Table 2, Fig. 2C); therefore, they were used to develop a risk calculator for bedside use (Fig. 3). Interestingly, in the logistic LASSO model, day 3 level of the CRP (among the first 3 days values) had the highest impact. However, in other models, the values on day 1–3 (the “trend” over the first 3 days) were important (Supplementary table S1), emphasizing the different algorithms used in the 5 prediction models.

Identifying patients with a hyper-inflammatory immune response early during the disease course may enable timely treatment of those at risk of high mortality. This is of particular interest since progression to acute respiratory distress syndrome or sepsis often marks the “point of no return”, where most treatment options (including high dose glucocorticoids) become ineffective^20^. Therefore, targeted blockade of additional detrimental hyper-inflammatory responses using early glucocorticoid and/or a monoclonal antibody (neutralizing proinflammatory IL-6) therapy might prevent exacerbation^21,22^. This can, optimally, facilitate allocation of limited resources during a pandemic (and prevent the collapse of the healthcare system); patients at a low risk can be discharged from hospital safely after 3 days of observation to self-quarantine at home or in a living and treatment center^7^, whereas patients at a high risk should remain in hospital for close monitoring and intensive treatment. Further studies are needed to investigate whether implementing the CHANeL model will save more lives and/or shorten hospital stay.

This study has several limitations. First, this study included only hospitalized Korean patients. External validation of the CHANeL models in different ethnic groups is required. Second, the mortality in this cohort was 2.7% whereas the current mortality of COVID-19 is 1.6% in Korea^23^. As the patients in this cohort only included hospitalized patients, the mortality was expected to be significantly higher than that in the general population, indicating that the relatively mild COVID-19 cases were included (58.2% of patients in the training set and 63.1% in the test set did not require any oxygen supplementation). This is, in part, due to the low incidence of COVID-19 in Korea, allowing the many patients with mild COVID-19 being treated as inpatients. The higher proportion of the non-O2 requirement, however, help to build the model better. Third, information on arterial blood gas analysis or PaO_2_/FiO_2_ (PF) ratio was not available in all patients. Instead, we utilized SpO_2_/FiO_2_ ratio which correlates with PF ratio^24^. Last but not the least, the primary aim of the study was to identify high risk patients who require more intensive monitoring and treatment (i.e. oxygen requirement as a surrogate marker for more severe disease). Therefore, an ideal study population would be patients who are just diagnosed with COVID-19. Accordingly, the prediction models need to be validated in a prospective cohort of patients who are diagnosed with COVID-19.

In conclusion, CHANeL prediction models based on serial measurements of CRP, ANC, and ALC during first 3 days of hospitalization, along with age and hypertension, provide an accurate estimate of the risk of supplement oxygen requirement among hospitalized patients with COVID-19. Further studies are needed to examine whether implementing this model at bedside can improve outcomes and shorten hospital stays.

## Methods

### Patients and data collection

This retrospective cohort study included COVID-19 patients who were treated at five medical centers designated for treatment of COVID-19 by the South Korean government. A diagnosis of COVID-19 was confirmed by a positive SARS-CoV-2 real-time reverse transcriptase–polymerase chain reaction result from a respiratory sample; RT-PCR was performed at the participating institutions or at the Korea Centers for Disease Control and Prevention. The cohort included 280 consecutive patients with COVID-19, all of whom were admitted to one of the five hospitals from January 24, 2020 through July 10, 2020. After excluding patients with incomplete information about medications, the patients were assigned randomly to a training set and a test set in a ratio of 1:1. Of note, the patients included in this study were the same as the patients included in our prior study^8^.

Demographic and laboratory data were obtained from electronic medical records. The study was conducted in accordance with the principles of the Declaration of Helsinki and Good Clinical Practice guidelines. The study was approved by the institutional review board of each participating center (NMC, SNUBH, SNUH, Armed Forces Capital Hospital, Myongji hospital). The institutional review board of each participating center (NMC, SNUBH, SNUH, Armed Forces Capital Hospital, Myongji hospital) waived informed consent because the study involved a minimum risk to the patient and no identifiable information was used.

### Outcome

The primary outcome was a requirement for supplementary oxygen during the hospitalization period. Supplementary oxygen requirement, ranging from nasal prongs to mechanical ventilation, is a marker of COVID-19 severity and an important indication for close monitoring and treatment. A previous study showed that all patients with COVID-19 who did not require supplementary oxygen had a mild disease course and a good prognosis^8^.

### Selection of CHANeL predictors

Two demographic variables (age and history of hypertension) were selected a priori; both of these are known risk factors for severe COVID-19 disease^25^. In addition, three routine inflammatory markers (CRP, absolute neutrophil count (ANC), and absolute lymphocyte count (ALC)) during the first 3 days of hospitalization were selected. Predictor selection was based on the previous observation that longitudinal patterns of CRP, ANC, and ALC are highly associated with a particular type of inflammatory response and clinical outcome, including oxygen requirement and death^8^.

Missing values were imputed using linear interpolation between the non-missing values immediately before and after the missing time point, with a calculated variation that follows the shape of the population’s average trajectory^26^. Patients for whom missing data could not be imputed reliably were excluded.

### Construction of prediction models

Logistic regression, logistic LASSO regression, Random Forest, Support Vector Machine, and XGBoost analysis were tested using the five CHANeL predictors. The ability of each model to predict supplementary oxygen requirement was assessed by calculating the area under the receiver-operator characteristic curve (AUC). A training set and a test set was used to test each model for sensitivity (proportion of oxygen requirement cases predicted correctly), specificity (proportion of no-oxygen requirement cases predicted correctly), and accuracy (proportion of cases predicted correctly).

### Statistical analysis

Continuous variables and categorical variables were compared using t-tests or the Mann–Whitney U-test, or the Chi-squared test or Fisher’s exact test, as appropriate. Statistical analysis was performed using RStudio (version 1.2; Boston, MA, USA) and SPSS (IBM SPSS Statistics for Windows, Version 25.0). A *P*-value < 0.05 was considered statistically significant.

## Supplementary Information

Supplementary Information.
