# Supplementary material for: Predication of oxygen requirement in COVID-19 patients using dynamic change of inflammatory markers: CRP, hypertension, age, neutrophil and lymphocyte (CHANeL)
Source: Sci Rep. 2021 Jun 22;11:13026. doi: 10.1038/s41598-021-92418-2 (PMC8219792; doi:10.1038/s41598-021-92418-2)
Supplement: Supplementary file 1 — Supplementary Information. [file 41598_2021_92418_MOESM1_ESM.docx]

*Supplementary information file*

**“Predication of oxygen requirement in COVID-19 patients using dynamic change of inflammatory markers: CRP, Hypertension, Age, Neutrophil and Lymphocyte (CHANeL)”**

Eunyoung Emily Lee, MD^a*^, Woochang Hwang, PhD^b*^, Kyoung-Ho Song, MD, PhD^c^, Jongtak Jung, MD^c^, Chang Kyung Kang, MD, PhD^d^, Jeong-Han Kim, MD^e^, Hong Sang Oh, MD, MPH^e^, Yu Min Kang, MD^f,g^, Eun Bong Lee, MD, PhD^h^, BumSik Chin, MD, PhD^i^, Woojeung Song^j^, Nam Joong Kim, MD, PhD^d**^, and Jin Kyun Park, MD, PhD^h**^

^*^Both authors contributed equally to the work.

^a^Division of Rheumatology, Department of Internal Medicine, Uijeongbu Eulji Medical Center, Eulji University School of Medicine, Gyeonggi-do, Korea, ^b^Hanyang Biomedical Research Institute, Hanyang University, Seoul, Korea, ^c^Division of Infectious Diseases, Department of Internal Medicine, Seoul National University Bundang Hospital, Seongnam, Korea, ^d^Division of Infectious Diseases, Department of Internal Medicine,

Seoul National University College of Medicine, Seoul, Korea, ^e^Division of Infectious Diseases, Department of Internal Medicine, Armed Forces Capital Hospital, Seongnam-Si, Gyeonggi-do, Korea, ^f^Department of Infectious Diseases, Myongji hospital, Gyeonggi-do, Korea, ^g^Department of Medical Education, Seoul National University College of Medicine, ^h^Division of Rheumatology, Department of Internal Medicine, Seoul National University College of Medicine, Seoul, Korea, ^i^Division of Infectious Diseases, Department of Internal Medicine, National Medical Center, Seoul, Korea, ^j^Department of Medicine, Major in Medical Genetics, Graduate School, Hanyang University, Seoul, Korea.

|  | CRP Day 1 | CRP Day 2 | CRP Day 3 | ANC Day 1 | ANC Day 2 | ANC Day 3 | ALC Day 1 | ALC Day 2 | ALC Day 3 | Age | Hypertension |
| --- | --- | --- | --- | --- | --- | --- | --- | --- | --- | --- | --- |
| Logistic Regression | 0.63 | -3.2 | 5.38 | -0.3 | -0.39 | 1.92 | -0.88 | 0.12 | 0.94 | 0.07 | -0.75 |
| Logistic Lasso Regression | 0 | 0 | 1.55 | 0 | 0 | 1.03 | 0 | 0 | 0.13 | 0.04 | 0 |
| Random Forest | 6.67 | 5.93 | 11.76 | 2.83 | 4.3 | 6.67 | 3.27 | 2.4 | 3.12 | 5.7 | 0.33 |
| Support Vector Machine | 7.78 | 8.4 | 13.08 | 2.54 | 8.16 | 11.42 | 4.02 | 1.66 | 0.65 | 11.79 | 0.76 |
| XGBoost | 0.005 | 0.029 | 0.31 | 0.002 | 0.0005 | 0.051 | 0.011 | 0.005 | 0.013 | 0.045 | 0.017 |

**Supplementary table S1**. Contribution of parameters to the prediction models

ALC, absolute lymphocyte count; ANC, absolute neutrophil count; CRP, C-reactive protein

**Supplementary table S2**. Modes of oxygen delivery

|  | Flow rate | FiO2 delivered | Number of patients | | |
| --- | --- | --- | --- | --- | --- |
|  |  |  | Total | Train set | Test set |
| Nasal cannula/ facial mask | 1L/min~6L/min, 6L/min~10L/min (facial mask) | 24-44%, 35-60% (facial mask) | 35 | 13 | 22 |
| HFNC |  | 0.4-0.9. | 28 | 17 | 11 |
| Invasive MV |  | 0.4-1.0 | 19 | 13 | 6 |
| ECMO |  | 0.4-1.0 | 5 | 3 | 2 |

ECMO, extracorporeal membrane oxygenation; HFNC, high flow nasal cannula; MC, mechanical ventilator
